# High Propensity for Multidrug-Resistant Pneumococcal Shedding Among Adults Living With HIV on Stable Antiretroviral Therapy in Malawi

**DOI:** 10.1093/ofid/ofaf422

**Published:** 2025-07-16

**Authors:** Lusako L Sibale, Newton Kalata, Ndaona Mitole, Tinashe K Nyazika, Joseph A Phiri, Alice Kusakala, Mercy Khwiya, Gift Sagawa, Stephanie W Lo, Chrispin Chaguza, Deus Thindwa, Todd D Swarthout, Neil French, Ken Malisita, Arox Kamng’ona, Daniela M Ferreira, Stephen D Bentley, Robert S Heyderman, Brenda A Kwambana-Adams, Kondwani C Jambo

**Affiliations:** Infection and Immunity Research Group, Malawi-Liverpool-Wellcome Programme, Blantyre, Malawi; Department of Clinical Sciences, Liverpool School of Tropical Medicine, Liverpool, UK; Parasites and Microbes, Wellcome Sanger Institute, Cambridge, UK; Infection and Immunity Research Group, Malawi-Liverpool-Wellcome Programme, Blantyre, Malawi; Infection and Immunity Research Group, Malawi-Liverpool-Wellcome Programme, Blantyre, Malawi; Department of Biomedical Sciences and Physiology, School of Life Sciences, Faculty of Science and Engineering, University of Wolverhampton, Wolverhampton, UK; Infection and Immunity Research Group, Malawi-Liverpool-Wellcome Programme, Blantyre, Malawi; Department of Clinical Sciences, Liverpool School of Tropical Medicine, Liverpool, UK; Infection and Immunity Research Group, Malawi-Liverpool-Wellcome Programme, Blantyre, Malawi; Infection and Immunity Research Group, Malawi-Liverpool-Wellcome Programme, Blantyre, Malawi; Infection and Immunity Research Group, Malawi-Liverpool-Wellcome Programme, Blantyre, Malawi; Parasites and Microbes, Wellcome Sanger Institute, Cambridge, UK; Milner Centre for Evolution, Department of Life Sciences, University of Bath, Bath, UK; The Great Ormond Street Institute of Child Health, University College London, London, UK; Research Department of Infection, Division of Infection and Immunity, University College London, London, UK; Institute of Infection Veterinary & Ecological Science, University of Liverpool, Liverpool, UK; Oxford Vaccine Group, University of Oxford, Oxford, UK; Department of Epidemiology of Microbial Diseases, Yale School of Public Health, Yale University, New Haven, Connecticut, USA; Infection and Immunity Research Group, Malawi-Liverpool-Wellcome Programme, Blantyre, Malawi; Department of Epidemiology of Microbial Diseases, Yale School of Public Health, Yale University, New Haven, Connecticut, USA; Research Department of Infection, Division of Infection and Immunity, University College London, London, UK; Institute of Infection Veterinary & Ecological Science, University of Liverpool, Liverpool, UK; Lighthouse, Queen Elizabeth Hospital and Gateway Health Centre, Blantyre District Health Office, Blantyre, Malawi; School of Life Science and Allied Health Professions, Kamuzu University of Health Sciences, Blantyre, Malawi; Department of Clinical Sciences, Liverpool School of Tropical Medicine, Liverpool, UK; Oxford Vaccine Group, University of Oxford, Oxford, UK; Parasites and Microbes, Wellcome Sanger Institute, Cambridge, UK; Research Department of Infection, Division of Infection and Immunity, University College London, London, UK; Infection and Immunity Research Group, Malawi-Liverpool-Wellcome Programme, Blantyre, Malawi; Department of Clinical Sciences, Liverpool School of Tropical Medicine, Liverpool, UK; Research Department of Infection, Division of Infection and Immunity, University College London, London, UK; Infection and Immunity Research Group, Malawi-Liverpool-Wellcome Programme, Blantyre, Malawi; Department of Clinical Sciences, Liverpool School of Tropical Medicine, Liverpool, UK; School of Life Science and Allied Health Professions, Kamuzu University of Health Sciences, Blantyre, Malawi

**Keywords:** AMR, ART, carriage, HIV, *Streptococcus pneumoniae*

## Abstract

**Background:**

People living with human immunodeficiency virus (HIV; PLHIV) on antiretroviral therapy (ART) are still at risk of pneumococcal disease and have over 2-fold higher pneumococcal carriage prevalence than HIV-uninfected (HIV−) adults). Carriage is a risk factor for pneumococcal disease, antimicrobial resistance (AMR) emergence, and transmission. Therefore, we tested whether the high prevalence of pneumococcal carriage in PLHIV on ART is associated with increased bacterial density, shedding, and AMR.

**Methods:**

We recruited asymptomatic PLHIV on ART for >1 year (PLHIV-ART>1y) and HIV− adults. Nasopharyngeal swab samples were collected on days 3, 7, 14, 21, and 28, followed by monthly collections for 12 months, while shedding samples were collected on days 3, 21, and 28. Peripheral blood samples were collected on day 3 to measure CD4 cell count and HIV viral load. Pneumococcal carriage density and shedding were assessed using standard bacterial culture, multiple carriage was detected using whole-plate sweep sequencing, and AMR profiling was conducted using disk diffusion and Etest.

**Results:**

PLHIV-ART>1y had a higher propensity for high-density carriage (adjusted odds ratio, 1.67 [95% confidence interval (CI), 1.07–2.60]; *P* = .02). Moreover, PLHIV-ART>1y are more likely to shed pneumococci than HIV− adults (adjusted odds ratio, 2.52 [95% CI, 1.06–6.00]; *P* = .04), with carriage density identified as an important risk factor for shedding (3.35 [1.55–7.24]; *P* = .002). Aerosol shed isolates from PLHIV-ART>1y were mostly multidrug resistant (18 of 29 [ 62%; 95% CI, 48%–77%]).

**Conclusions:**

These findings indicate that PLHIV-ART>1y remain at high risk of pneumococcal disease and could also be an important reservoir for shedding multidrug-resistant pneumococci.

People living with human immunodeficiency virus (HIV; PLHIV) and receiving antiretroviral therapy (ART) are at higher risk of invasive pneumococcal disease (IPD) but also have >2-fold prevalence of pneumococcal carriage compared with HIV-uninfected (HIV−) adults. Persistent pneumococcal carriage is a risk factor for emergence and transmission of antimicrobial resistance (AMR) , but it remains unclear whether PLHIV on ART could be a potential reservoir of multidrug-resistant (MDR) pneumococcal transmission.

The current study demonstrates that PLHIV on long-term ART not only remain at risk of IPD but also harbor and commonly shed MDR pneumococci. Its findings support a reevaluation of the provision of pneumococcal vaccination to this vulnerable adult population of PLHIV on stable ART, highlighting that suppressive ART alone is not sufficient to eliminate the risk of IPD in PLHIV from high-transmission and disease-burdened settings.

Pneumococcal carriage is a prerequisite for life-threatening IPD, including pneumonia, meningitis, and bacteremia [1], and it is critical for transmission [2]. Moreover, high pneumococcal carriage density is associated with IPD [3, 4]. In high- and middle-income countries, infant pneumococcal conjugate vaccine (PCV) programs have significantly reduced pneumococcal carriage and IPD in both vaccinated children (direct protection) and unvaccinated older children and adults (indirect protection) [5, 6]. However, in Malawi, a low-income country, where 13-valent PCV (PCV13) was introduced into the infant immunization program in 2011, there is limited indirect protection among unvaccinated older children and adults despite evidence of substantial direct protection against IPD among vaccinated children [7]. Furthermore, the residual carriage of all PCV13 serotypes among adults living with HIV on ART has remained relatively high [8, 9], with low socioeconomic status shown to exacerbate carriage prevalence [10].

In Malawi, pneumococcal carriage prevalence is 2-fold higher in PLHIV on ART (40%–60%) than in HIV− individuals (8%–15%) or ART-naive PLHIV (18%–25%) [8, 11]. Pneumococcal carriage is an important risk factor for AMR emergence [12, 13]. Common viral coinfections promote high pneumococcal carriage density that drives increased bacterial shedding [14, 15], a surrogate for transmission potential [2, 16], are common in PLHIV [17–20] Together, this may suggest that PLHIV could be an underappreciated reservoir of AMR pneumococcal transmission in the community, calling for an in-depth evaluation of this population.

Given the knowledge gap related to the public health relevance of high pneumococcal carriage in PLHIV on ART, we conducted a study to test whether the high pneumococcal carriage prevalence in PLHIV on ART is associated with increased bacterial density, shedding and AMR. The study has implications for the development of interventions to reduce the risk of IPD in PLHIV and curb AMR emergence and transmission.

## METHODS

### Study Design and Recruitment

We recruited asymptomatic PLHIV on ART for >1 year (PLHIV-ART>1y) and HIV− adults in Blantyre, Malawi. PLHIV-ART>1y were enrolled during routine visits at ART clinics of Lighthouse–Queen Elizabeth Central Hospital, while HIV− adults were recruited from Voluntary Counselling and Testing centre at Gateway Health Centre during voluntary testing. Participants were enrolled on day 3 after screening and then followed up on days 7, 14, 21, and 28 for the first month and then every month for 12 months (Supplementary Figure 1).

Eligible participants were aged 18–45 years, confirmed pneumococcal carriers during screening (pneumococcal carriage was assessed using WHO-recommended nasopharyngeal sampling and standard microbiological culture [21]), living with a child ≤5 years old, and providing written informed consent. Exclusion criteria included antibiotic use within the past 4 weeks (except cotrimoxazole prophylaxis, which PLHIV received per national guidelines [22]), recent pneumonia-related hospitalization, respiratory Kaposi sarcoma, or terminal illness. All PLHIV were on standardized ART per Malawi's 2018 HIV management guidelines [23].

### Ethical Approval

The study was conducted following good clinical practice guidelines and the Declaration of Helsinki. Ethical approval was obtained from the College of Medicine Research Ethics Committee (no. P.11/18/2532) and Liverpool School of Tropical Medicine Research Ethics Committee (no. 19-033).

### Demographic Data

Demographic data were collected digitally using Open Data Kit (ODK Collect v2025.1.2) on mobile devices to enable real-time entry and improve accuracy. Information was self-reported and included age, sex, recent antibiotic use, ART initiation date and regimen, and household details such as the number and ages of children ≤5 years old. Socioeconomic status was assessed using a possession index based on ownership of 15 functional items [10]: watch, radio, bank account, iron (charcoal), sewing machine (electric), mobile phone, CD player, fan (electric), bed net, mattress, bed, bicycle, motorcycle, car, and television.

### Sample Collection

Nasopharyngeal swab samples were collected at all time points, and peripheral blood was collected at recruitment (day 3) for measuring CD4 cell counts and HIV viral loads (Supplementary Figure 1). Respiratory secretions were sampled on days 3, 21, and 28 to assess pneumococcal shedding, using a modified polyvinyl alcohol (PVA) face mask, direct coughing onto agar, and nose poking (Supplementary Figure 1). Nylon flocked nasopharyngeal swabs (Copan Diagnostics) were placed in skim milk–tryptone-glucose-glycerol (STGG) medium immediately after collection.

For face mask samples, participants wore a modified mask containing four 1 × 9-cm 3-dimensional–printed PVA strips [24–26] for 15 minutes, coughing every 5 minutes while engaged in conversation with clinical staff. For nose poking, participants inserted a clean index finger into the nose; the finger was swabbed (using a wooden cotton swab) and the swab was placed in STGG. For coughing samples, participants coughed directly onto gentamicin–sheep blood agar (SBG; 5% sheep blood and 5 μL/mL gentamicin). All samples were delivered to the lab

oratory within an hour, processed, and stored in STGG at −80°C. Shedding routes were categorized as mechanical (nose poking), aerosol (coughing), or face mask based. Pneumococcal shedding sample distribution and prevalence by specimen type are shown in Supplementary Figure 2.

### Microbiological Culture

Standard microbiological culture [21] was used to detect *Streptococcus pneumoniae* from nasopharyngeal and shedding samples. For modified PVA face mask processing, 4 PVA strips were aseptically removed, dissolved in 10 mL of Todd Hewitt broth with 5% yeast, and plated on an SBG. Colonies were identified based on morphology and optochin sensitivity after an overnight incubation at 37°C in 5% carbon dioxide (CO_2_). Plates with no growth were reincubated for an additional 24 hours before being reported negative. Isolates with no or intermediate optochin sensitivity (<14-mm zone) were confirmed using the bile solubility test. A single confirmed pneumococcal colony was subcultured for further analysis.

Serotyping was performed using latex agglutination (ImmuLex 23-valent Pneumotest; Statens Serum Institute), which differentiates PCV13 vaccine serotypes (vaccine type [VT]) but not nonvaccine serotypes (non-VT [NVT]); nontypeables were classified as NVT. Pneumococcal density was determined by serial dilution [27] culture on SBG and reported as colony-forming units (CFUs) per milliliter.

### AMR Profiling

The antimicrobial susceptibility of pneumococcal isolates was assessed using the disk diffusion method (Oxoid), oxacillin (1 μg), erythromycin (15 μg), tetracycline 3(0 μg), and cotrimoxazole (1.25–23.75 μg). Pure isolates were cultured on sheep blood agar overnight at 37°C in 5% CO_2_, then suspended in saline to match a 0.5 McFarland turbidity standard. The suspension was spread on Mueller-Hinton blood agar, and antibiotic discs were applied.

β-Lactam susceptibility was confirmed by Etest minimum inhibitory concentrations for benzylpenicillin. Colonies were suspended, adjusted to 0.5 McFarland, and plated on Mueller-Hinton blood agar, and Etest strips were applied per manufacturer's instructions. Plates were incubated overnight at 37°C in 5% CO₂, and minimum inhibitory concentrations were read at the intersection of growth inhibition with the strip.

Interpretation followed European Committee on Antimicrobial Susceptibility Testing (EUCAST) meningitis breakpoints [28]. *S pneumoniae* American Type Culture Collection 49619 was used as a quality control strain. MDR was defined as nonsusceptibility to ≥3 antibiotic classes.

### DNA Extraction, Sequencing, Serotype Calls and Species Abundance Calls

The shedding arm (paired nasopharyngeal and coughing samples in STGG, confirmed pneumococcus positive by standard culture) was analyzed using whole-genome sequencing to examine serotype distribution, multiple carriage, and species diversity. For plate sweep cultures, 100 μL of stored samples were plated onto Columbia CNA agar containing 5% sheep blood. These were then incubated overnight at 37°C and 5% CO_2_. All growth was collected using 10 μL of sterile plastic loops. DNA was extracted using the QIAamp DNA Mini Kit (Qiagen) per the manufacturer's protocol and quantified with an Invitrogen Qubit fluorometer. Samples with >2.5 μg of DNA were submitted for sequencing.

Paired nasopharyngeal and cough sweeps were sequenced on the Illumina NovaSeq 6000 platform (SP flow cell; 150-base pair paired-end reads, 384-plex, approximately 1.8 million reads per sample). Serotype composition was determined using SeroCall [29], a bioinformatics tool for identifying pneumococcal serotypes, including mixed carriage. Species composition was assessed using the Kraken metagenomic classification algorithm (version 1.0.0) [30] with the standard database.

### Statistical Analysis

Descriptive statistics are reported as numbers and proportions for categorical variables, and medians with 95% confidence intervals (CIs) for continuous variables. Categorical variables were compared using the χ^2^ test, and continuous variables using the Wilcoxon rank sum test. To account for repeated measures, generalized linear mixed models (GLMMs) with a nested random effect for participant and visit day were used.

GLMMs were used to examine factors associated with 3 outcomes: VT versus NVT carriage, high-density pneumococcal carriage, and pneumococcal shedding. Each model included the relevant outcome as the dependent variable and HIV status as the primary exposure. Due to the low prevalence of recent antibiotic use at the time of sampling (2.4% in PLHIV-ART > 1 year and 5.7% in HIV− adults), this variable was excluded from final models. Sensitivity analysis excluding these samples confirmed that this exclusion had no impact on the main findings. Full model covariates and variable definitions are provided in the Supplementary materials.

All data analyses were performed using RStudio software (version 4.1.3; R Development Core Team). Figures and tables were produced using R (version 4.1.3), RStudio (version 2022.02. 2+485.pro2 Prairie Trillium; 27 April 2022), ggplot2 (version 3.3.5), lme4 (version 1.1-28), and gtsummary (version 1.5.2) software. Differences were considered statistically significant at *P* < .05.

## RESULTS

### Clinical and Demographic Characteristics

**Figure 1.**
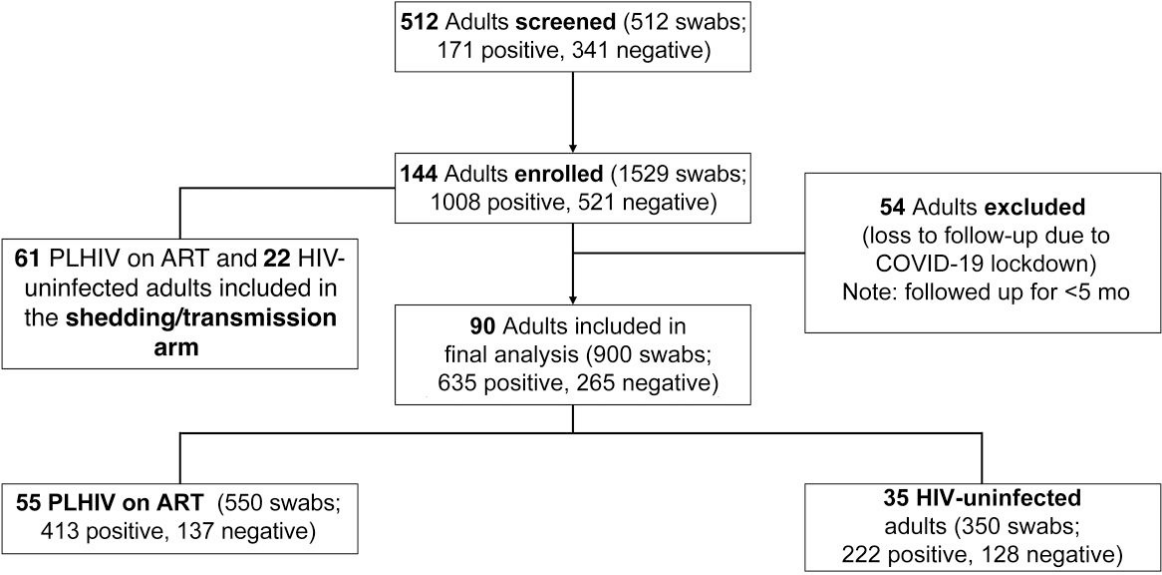
Recruitment flow diagram showing the number of adults and pneumococcal samples included in the analysis among people living with human immunodeficiency virus (HIV; PLHIV). Abbreviations: ART, antiretroviral therapy; COVID-19, coronavirus disease 2019.

Between July 2019 and August 2021, we screened 512 adults, among whom 28% (144 of 512) with confirmed pneumococcal carriage were recruited (Figure 1). Of these, 37.5% (54 of 144) were HIV− adults and 62.5% (90 of 144) were PLHIV-ART>1y (median ART duration [interquartile range (IQR)], 5.5 [2.8–10.1] years) (Supplementary Table 1). The study encountered challenges with participant retention due to the coronavirus disease 2019 pandemic. To address this issue, we focused the pneumococcal carriage prevalence and density analysis on participants who completed 5 months of follow-up visits (62.5% [90 of 144]) (Figure 1). Therefore, the analysis was restricted to the 35 HIV− adults and 55 PLHIV-ART>1y (Figure 1). However, for the shedding arm the analysis was based on 22 HIV− adults and 61 PLHIV-ART>1y who were recruited (Figure 1 and Supplementary Figure 2). Compared with HIV− adults, PLHIV-ART>1y were older, with median ages (IQR) of 34 (28–39) and 27 (23–35) years, respectively (*P* = .01) (Table 1). Likewise, compared with HIV− adults, PLHIV-ART>1y had lower socioeconomic status (median score [IQR], 4 [2.0–5.0] vs 6 [3.5–7.50], respectively; *P* = .005) and a lower CD4 cell count (median, 514/μL [652–927/μL] vs 760/μL [332–750/μL]; *P* < .001) (Table 1).

**Table 1.** Baseline Characteristics of Participants Who Completed ≥5 Months of Follow-up

Characteristic	HIV− (n = 35)	PLHIV-ART>1y (n = 55)	*P* Value
Sex, no. (%)			.7^a^
Female	25 (75)	41 (75)	
Male	10 (29)	14 (25)	
Age, median (IQR), y	27 (23–35)	34 (28–39)	**.01^b,c^**
No. of children aged ≤5 y in household, no. (%)			.7^a^
1	26 (74)	45 (82)	
2	8 (23)	9 (16)	
3	1 (2.9)	1 (1.8)	
SES score, median (IQR)^d^	6 (3.5–7.5)	4 (2–5)	**.005^b,c^**
CD4 cell count, median (IQR), cells/μL	760 (652–927)	514 (332–750)	**<.001^b,c^**
ART duration, median (IQR), y	NA	5.8 (3.4–11.3)	…
HIV viral load, median (IQR), copies/mL^e^	NA	39 (39–18 928)	…

Abbreviations: ART, antiretroviral therapy; HIV, human immunodeficiency virus; IQR, interquartile range; NA, not applicable; PLHIV-ART>1y, people living with HIV and on ART for >1 year; SES, socioeconomic status.

^a^ *P* value based on Pearson χ^2^ or Fisher exact test.

^b^ *P* value based on Wilcoxon rank sum test.

^c^Significant at *P* < .05.

^d^The SES score is based on a possession index, calculated as a sum of positive responses for household ownership of each of 15 functioning items: a watch, radio, bank account, iron (charcoal), sewing machine (electric), mobile phone, CD player, fan (electric), bed net, mattress, bed, bicycle, motorcycle, car, and television.

^e^Only 10 PLHIV-ART>1y had a detectable viral load.

### NVTs as Main Drivers of High Pneumococcal Carriage Prevalence in PLHIV-ART>1y

**Figure 2.**
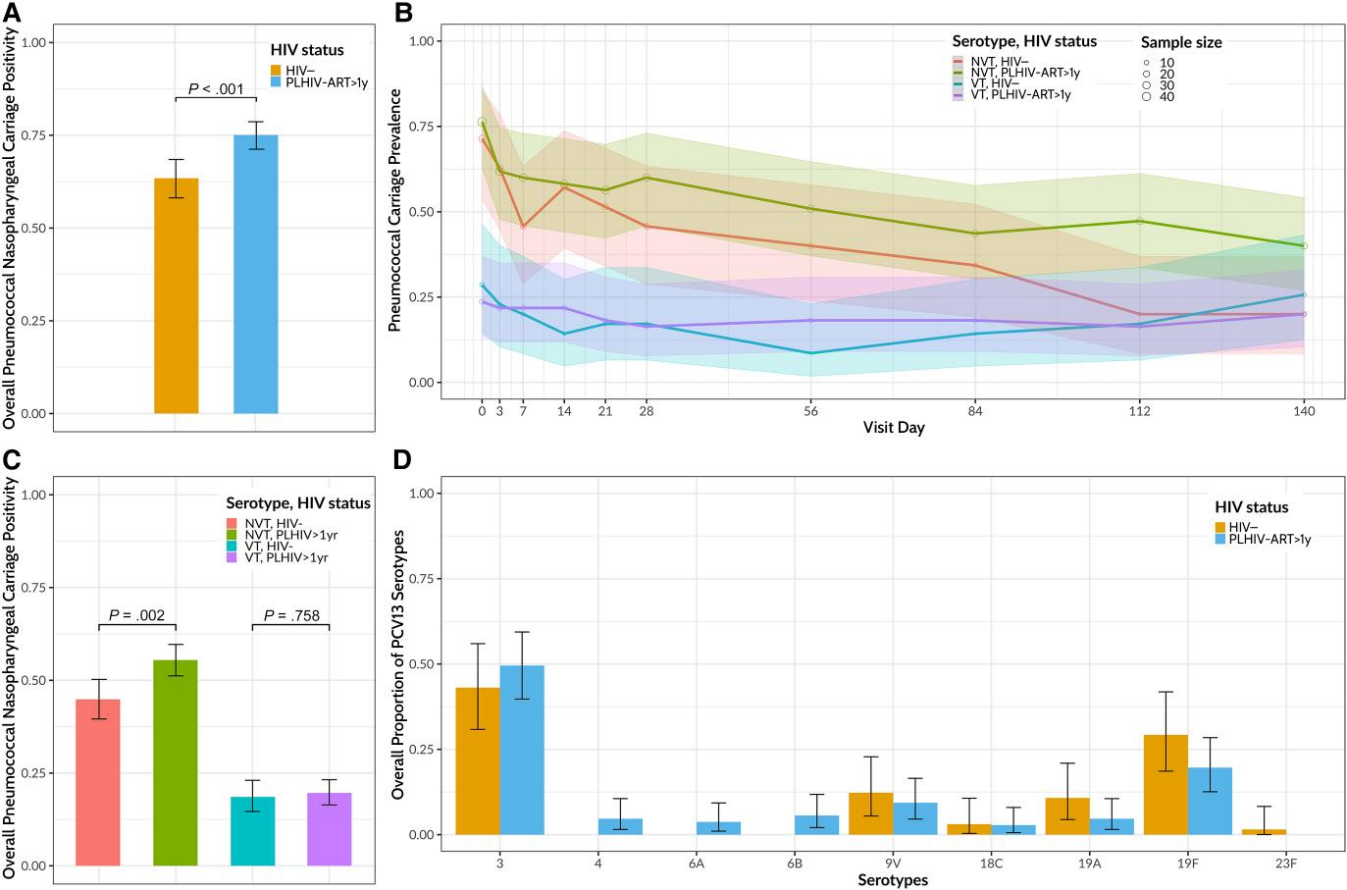
Overall phenotypic pneumococcal swab positivity among people living with human immunodeficiency virus (HIV) and on antiretroviral therapy for >1 year (PLHIV-ART>1y) and HIV-uninfected (HIV−) adults, including 13-valent pneumococcal conjugate vaccine (PCV13) serotypes (vaccine type [VT]) and non-PCV13 serotypes (non-VT [NVT]). *A*, HIV-stratified pneumococcal swab sample positivity. *B*, HIV- and serogroup-stratified pneumococcal swab sample positivity by nasopharyngeal sampling visit. *C*, HIV- and serogroup-stratified pneumococcal swab sample positivity. *D*, HIV-stratified proportion of PCV13 serotypes. The denominator for each serotype is the total number of PCV13 serotypes in each group. Whiskers represent 95% confidence intervals. Data were analyzed using χ^2^ tests (35 HIV− adults; 55 PLHIV-ART>1y). Source data are provided as a Source Data file.

Overall nasopharyngeal swab pneumococcal positivity was higher among PLHIV-ART>1y compared with HIV− adults (413 of 550 [75.1%; 95% CI, 68.2%–76.2%] vs 222 of 350 [63.4%; 53.7%–64.8%], respectively; *P* < .001) (Figure 2*A*). To assess the indirect impact of children immunization with PCV13 in adults, also referred to as “herd immunity,” VT and NVT carriage were disaggregated in the subsequent analysis. NVT nasopharyngeal swab pneumococcal positivity was higher in PLHIV-ART>1y than in HIV− adults (305 of 550 [55.5%; 95% CI, 51.2%–59.7%] vs 157 of 350 [44.9%; 39.6%–50.2%], *P* = .002) (Figure 2*B* and 2*C*). In contrast, VT nasopharyngeal swab pneumococcal positivity did not differ significantly between PLHIV-ART>1y and HIV− adults (108 of 550 [19.6%; 95% CI, 16.4%–23.2%] and 65 of 350 [18.6%; 14.6%–23.0%], respectively; *P* = .58) (Figure 2*B* and 2*C*). The dominant VT carriage pneumococci among both PLHIV-ART>1y and HIV− adults were serotype 3 (49.1% [53 of 108] and 43.1% [28 of 65]) and serotype 19F (19.4% [21 of 108] and 29.2% [19 of 65]) (Figure 2*D*).

Using a multivariable logistic regression model, we showed that PLHIV-ART>1y were more likely to carry NVT than HIV− adults (adjusted odds ratio [aOR], 1.45 [95% CI, 1.10–1.93]; *P* = .009) (Supplementary Table 2a). In contrast, the likelihood of having VT carriage was similar between PLHIV-ART>1y and HIV− adults (aOR, 17.0 [95% CI, 0.07–4095]; *P* = .3) (Supplementary Table 2b). Together, these data reveal that the high pneumococcal carriage prevalence in PLHIV-ART>1y is mostly driven by NVT. It also shows high residual VT carriage of serotypes 3 and 19F in this adult population.

### Higher and More Sustained Pneumococcal Carriage Density in PLHIV-ART>1y Than in HIV− Adults

**Figure 3.**
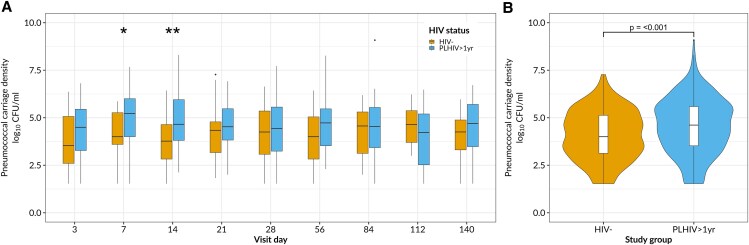
Pneumococcal carriage density among people living with human immunodeficiency virus (HIV) and on antiretroviral therapy for >1 year (PLHIV-ART>1y) and HIV-uninfected (HIV−) participants. *A*, Log median carriage density stratified by HIV status for 5 months of study follow-up. *B*, Aggregated log median carriage density stratified by HIV status for 5 months of study follow-up. For all box plots, box boundaries correspond to 25th and 75th percentiles; whiskers extend to a maximum of 1.5 times the interquartile range, with values outside the box and whiskers being outliers. Data were analyzed using the Wilcoxon test (35 HIV− adults [187 swab samples]; 55 PLHIV-ART>1y [358 swab samples]). Pneumococcal density was not assessed on screening samples or on day 0. *P = .04; **P = .006. Abbreviation: CFUs, colony-forming units.

Next, we sought to determine whether pneumococcal carriage density was different between the PLHIV-ART>1y and HIV− groups throughout the follow-up period. We observed a consistent trend of higher carriage density among PLHIV-ART>1y. Notably, this difference reached statistical significance at days 7 and 14, indicating a clear divergence in density trajectories during early follow-up. At day 7, the median (IQR) log₁₀ carriage density among HIV− adults was 4.00 ( 3.52–5.52) log₁₀ CFUs/mL, compared with 5.22 (4.00–6.00) log₁₀ CFUs/mL in PLHIV-ART>1y (*P* = .04). On day 14, the median (IQR) log₁₀ density in HIV− adults was 3.77 (2.83–4.64) log₁₀ CFUs/mL, while in PLHIV-ART>1y it was 4.66 (3.79–5.96) log₁₀ CFUs/mL (*P* = .006) (Figure 3*A*).

When data from all-time points were aggregated, the overall median log₁₀ pneumococcal density (IQR) remained significantly higher in PLHIV-ART>1y than in HIV− adults (4.61 [3.53–5.59] vs 4.00 [3.13–5.13] log₁₀ CFUs/mL, respectively; *P* < .001) (Figure 3*B*). Furthermore, in a multivariable logistic regression model, PLHIV-ART>1y were more likely to harbor higher density carriage than HIV-participants (aOR, 1.67, [95% CI, 1.07–2.60]; *P* = .02) (Table 2). These data demonstrate not only a higher baseline propensity for high-density pneumococcal carriage among PLHIV-ART>1y but also sustained elevated density over time.

**Table 2.** Factors Associated With Pneumococcal Carriage Density

Factor	No. With High Pneumococcal Density^a^/Total No.	Univariate Analysis		Multivariable Analysis	
		OR (95% CI)	*P* Value	OR (95% CI)	*P* Value
HIV status					
HIV−	130/187	1.52 (1.02–2.27)	**.04^b^**	1.67 (1.07–2.60)	**.02^b^**
PLHIV-ART>1y	278/358				
Sex					
Female	305/405	0.91 (.59–1.41)	.7	0.85 (.54–1.35)	.5
Male	103/140				
Age (y)	408/545	0.99 (.96–1.02)	.5	0.97 (.95–1.00)	.1
Season					
Cold dry	163/211	0.81 (.54–1.21)	.3	0.75 (.50–1.15)	.2
Hot wet	244/333				
SES score^c^					
Medium/high (>3)	245/333	1.19 (.81–1.79)	.4	1.15 (.75–1.75)	.5
Low (≤3)	163/212				

Abbreviations: CI, confidence interval; HIV, human immunodeficiency virus; HIV−, HIV uninfected; OR, odds ratio; PLHIV-ART>1y, people living with HIV and on antiretroviral therapy for >1 year; SES, socioeconomic status.

^a^High pneumococcal density was defined as >2010 colony-forming units (CFUs)/mL; low density, as ≤2010 CFUs/mL.

^b^Significant at *P* < .05.

^c^The SES score is based on a possession index, calculated as a sum of positive responses for household ownership of each of 15 functioning items: a watch, radio, bank account, iron (charcoal), sewing machine (electric), mobile phone, CD player, fan (electric), bed net, mattress, bed, bicycle, motorcycle, car, and television.

### Higher Proportion of Pneumococcal Shedding in PLHIV-ART>1y Than in HIV− Adults

**Figure 4.**
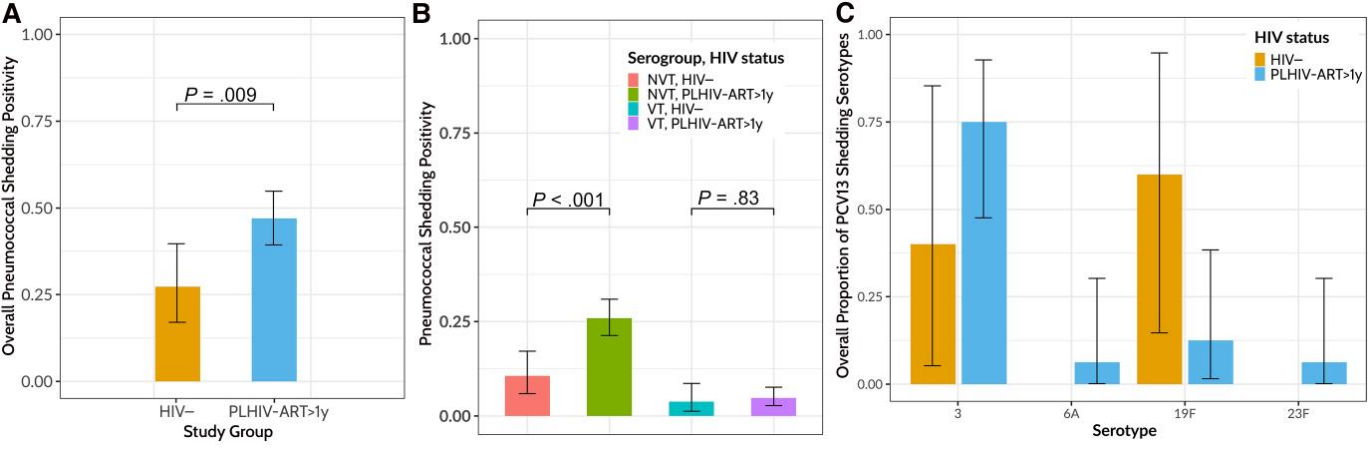
Phenotypic pneumococcal shedding among people living with human immunodeficiency virus (HIV) and on antiretroviral therapy for >1 year (PLHIV-ART>1y) and HIV-uninfected (HIV−) adults. Pneumococcal shedding was defined as the presence of viable pneumococci following microbiological culture of samples collected through nose poking (mechanical) and coughing (aerosol) or face mask sampling. Viable pneumococci were isolated in only 3 PLHIV-ART>1y using the face mask approach. *A*, Overall pneumococcal shedding positivity stratified by HIV status. *B*, Overall pneumococcal shedding positivity stratified by HIV status and serogroup. *C*, Overall pneumococcal shedding positivity stratified by HIV status and 13-valent pneumococcal conjugate vaccine (PCV13) serotypes; the denominator for each serotype is the total number of PCV13 serotypes in each group. Bars represent medians and whiskers, confidence intervals. Data were analyzed using χ^2^ tests (22 HIV− adults; 61 PLHIV-ART>1y). Source data are provided as a Source Data file.

Furthermore, considering the high-density carriage in PLHIV-ART>1y, we sought to determine whether they were also more likely to shed pneumococci than HIV− adults. The overall proportion of adults shedding pneumococci irrespective of sampling technique was significantly higher among PLHIV-ART>1y than in HIV− adults (79 of 168 [47%; 95% CI, 39.3%–54.9%] vs 18 of 66 [27.2%; 17%–39.6%], respectively; *P* = .009) (Figure 4*A*). Moreover, NVTs were shed more frequently in PLHIV-ART>1y than in HIV− adults (87 of 336 [25.9%; 95% CI, 21.3%–30.9%] vs 14 of 132 [10.1%; 5.9%–17.2%]; *P* < .001) (Figure 4*B*). However, VT shedding was similar between PLHIV-ART>1y and HIV− adults (16 of 336 [4.8%; 95% CI, 2.7%–7.6%] vs 5 of 132 [3.8%; 1.2%–8.6%], *P* = .83) (Figure 4*B*). Among the shed VTs, serotype 3 (in 12 of 16 PLHIV-ART>1y [75%] and 2 of 5 HIV− adults [40%]) and serotype 19F (in 2 of 16 [12.5%] and 3 of 5 [60%], respectively) were dominant in both groups (Figure 4*C*).

Using a multivariable logistic regression model, we showed that HIV status and pneumococcal carriage density were significantly associated with pneumococcal shedding (Table 3). Specifically, PLHIV-ART>1y were twice as likely as HIV− adults to shed pneumococci (aOR 2.52 [95% CI, 1.06–6.00]; *P* = .04), whereas individuals with high-density pneumococcal carriage, irrespective of HIV status, were 3 times more likely to shed pneumococci than those with low-density carriage (aOR, 3.35 [95% CI, 1.55–7.24]; *P* = .002). Taken together, these findings show that PLHIV-ART>1y are more likely to shed pneumococci than HIV− adults and that pneumococcal density is an important risk factor for shedding.

**Table 3.** Factors Associated With Pneumococcal Shedding

Factor	No. With Pneumococcal Shedding^a^/Total No.	Univariate Analysis		Multivariable Analysis	
		OR (95% CI)	*P* Value	OR (95% CI)	*P* Value
HIV status					
HIV−	18/66	2.37 (1.16–6.75)	**.007^b^**	2.52 (1.06–6.00)	**.04^b^**
PLHIV-ART>1y	79/168				
Sex					
Female	62/145	0.87 (.51–1.49)	.6	1.19 (.58–2.45)	.6
Male	35/89				
Age (y)	97/234	1.03 (.99–1.07)	.13	1.03 (1.03–1.09)	.2
Season					
Cold dry	34/93	1.40 (.82–2.40)	.2	1.14 (.56–2.33)	.7
Hot wet	63/141				
SES score^c^					
Medium/high (> 3)	63/135	0.60 (.35–1.02)	.06	0.63 (.31–1.29)	.2
Low (≤3)	34/99				
NP carriage density					
Low (≤2010 CFUs/mL)	18/59	3.37 (1.73–6.57)	**<.001^b^**	3.35 (1.55–7.24)	**.002^b^**
Medium/high (>2010 CFUs/mL)	68/114				

Abbreviations: CFUs, colony-forming units; CI, confidence interval; HIV, human immunodeficiency virus; HIV−, HIV uninfected; NP, nasopharyngeal; OR, odds ratio; PLHIV-ART>1y, people living with HIV and on antiretroviral therapy for >1 year; SES, socioeconomic status.

^a^Pneumococcal shedding was defined as the presence of viable pneumococci following microbiological culture of samples collected through nose poking (mechanical) and coughing (aerosol) or face mask sampling.

^b^Significant at *P* < .05.

^c^The SES score is based on a possession index, calculated as a sum of positive responses for household ownership of each of 15 functioning items: a watch, radio, bank account, iron (charcoal), sewing machine (electric), mobile phone, CD player, fan (electric), bed net, mattress, bed, bicycle, motorcycle, car, and television.

### Shedding of AMR Pneumococci in PLHIV-ART>1y

Pneumococcal carriage is an important risk factor for AMR emergence [12, 13]. We assessed the antimicrobial susceptibility profile of the nasopharyngeal and shed isolates from PLHIV-ART>1y and HIV− adults. Overall, the antimicrobial susceptibility profile of nasopharyngeal carriage isolates was similar between these groups (Table 4). However, the proportion of baseline cotrimoxazole-resistant nasopharyngeal carriage isolates was significantly higher in PLHIV-ART>1y than in HIV− adults (50 of 51 [98%; 95% CI, 88%–100%] vs 26 of 34 [76%; 58%–89%], respectively; *P* = .002) (Table 4).

**Table 4.** Baseline Antibiogram of Pneumococcal Carriage Isolates

Susceptibility	HIV− Adults, No. (%) (n = 34)	PLHIV-ART>1y, No. (%) (n = 51)	*P* Value^a^
Cotrimoxazole			
Susceptible	8 (24)	1 (20)	**.002^b^**
Nonsusceptible	26 (76)	50 (98)	
Oxacillin			
Susceptible	14 (41)	26 (51)	.4
Nonsusceptible	20 (59)	25 (49)	
Tetracycline			
Susceptible	16 (47)	32 (63)	.2
Nonsusceptible	18 (53)	19 (37)	
Erythromycin			
Susceptible	21 (62)	41 (80)	.06
Nonsusceptible	13 (38)	10 (20)	

Abbreviations: HIV, human immunodeficiency virus; HIV−, HIV uninfected; PLHIV-ART>1y, people living with HIV and on antiretroviral therapy for >1 year.

^a^ *P* values based on Pearson χ^2^ and Fisher exact tests.

^b^Significant at *P* < .05.

In a pairwise analysis, the overall proportion of nonsusceptible tetracycline, benzylpenicillin, and MDR isolates were significantly higher in aerosol shed isolates than in nasopharyngeal carriage isolates from PLHIV-ART>1y (tetracycline, 19 of 29 [66%; 95% CI 46%–75%] vs 9 of 29 [31%; 14%–41%, respectively; *P* = .009]; benzylpenicillin, 18 of 29 [62%; 46%–75%] vs 9 of 29 [31%; 19%–48%; *P* = .02]; and MDR, 18 of 29 [62%; 48%–77%] vs 9 of 29 [31%; 17%–45%; *P* = .02]) (Table 5). In contrast, the AMR profile of mechanical shed isolates was similar to that of nasopharyngeal carriage isolates (Table 5). Among HIV− adults, no differences were observed in the antimicrobial susceptibility profile between shed and nasopharyngeal isolates, irrespective of the shedding route (Supplementary Table 3). Collectively, these data show that aerosol shed isolates from PLHIV-ART>1y were more likely MDR than nasopharyngeal carriage or mechanical shed isolates.

**Table 5.** Pneumococcal Antibiogram Comparing Nasopharyngeal Carriage and Shed Pneumococci in the Same Individual, Stratified by Sample Type Among People Living With HIV and Receiving Antiretroviral Therapy for >1 Year

	Aerosol Shedding vs NP Carriage, No. (%)			Mechanical Shedding vs NP Carriage, No. (%)		
	Aerosol Shedding (n = 29)	NP Carriage (n = 29)	*P* Value^a^	Mechanical Shedding (n = 39)	NP Carriage (n = 39)	*P* Value^a^
Cotrimoxazole	28 (97)	29 (100)	>.9	38 (97)	35 (90)	.4
Benzylpenicillin^b^	18 (62)	9 (31)	**.02^c^**	13 (33)	9 (23)	.3
Tetracycline	19 (66)	9 (31)	**.009^c^**	9 (23)	11 (28)	.6
Erythromycin	10 (34)	8 (28)	.6	7 (18)	10 (26)	.4
MDR	18 (62)	9 (31)	**.02^c^**	8 (21)	10 (26)	.6

Abbreviations: HIV, human immunodeficiency virus; MDR, multidrug resistance; NP, nasopharyngeal.

^a^ *P* values based on Pearson χ^2^ and Fisher exact tests.

^b^Minimum inhibitory concentration using European Committee on Antimicrobial Susceptibility Testing (EUCAST) meningitis breakpoints.

^c^Significant at *P* < .05.

### Microbial Diversity in Shedding Versus Nasopharyngeal Samples

**Figure 5.**
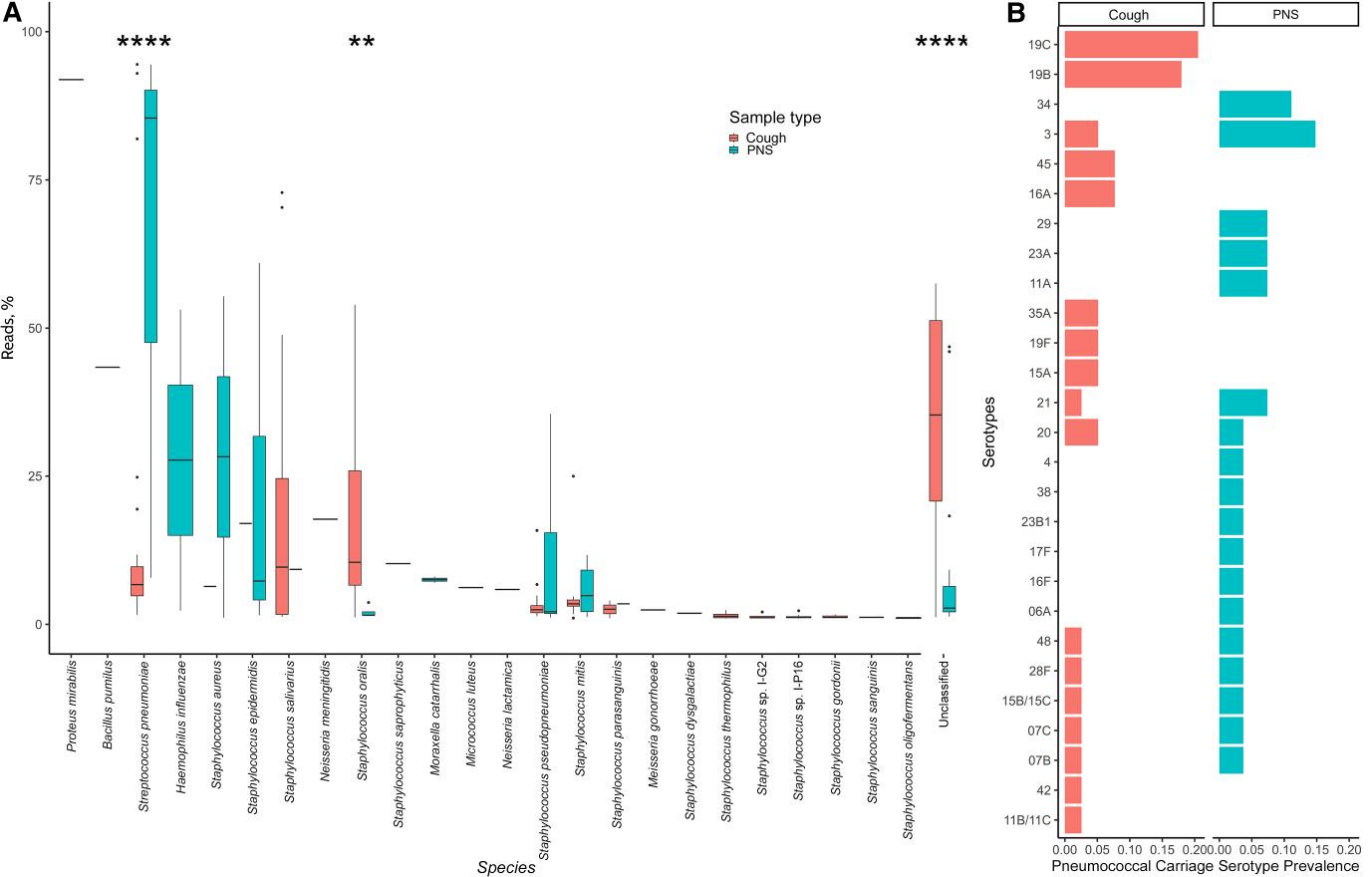
Species and pneumococcal serotype diversity from aerosol shedding (cough) and nasopharyngeal carriage (Posterior Nasopharyngeal Swab, PNS) among people living with human immunodeficiency virus and on antiretroviral therapy for >1 year (PLHIV-ART>1y) based on genomic data. *A*, Proportion of reads for each species per sample stratified by species and sample type. For all box plots, box boundaries correspond to 25th and 75th percentiles; whiskers extend to a maximum of 1.5 times the interquartile range, with values outside the box and whiskers being outliers. *B*, Proportions of serotypes stratified by serotype and sample type. These samples are pairs of PNS and cough isolates from the same individual among PLHIV-ART>1y. Data were analyzed using the Wilcoxon test (17 PLHIV-ART>1y; 19 nasopharyngeal swab and 19 aerosol samples). **P = .001; ****P < .001.

Studies on the drivers of pneumococcal virulence and AMR have postulated that closely related oral *Streptococcus* species are potential donors of virulent and AMR determinants [31, 32]. We assessed microbial diversity in paired nasopharyngeal and aerosol samples from PLHIV-ART>1y to explore the higher propensity for shedding and MDR pneumococci in aerosol isolates. Aerosol samples showed a higher abundance than nasopharyngeal samples for both unclassified species (median [IQR], 35.36% [20.78%–51.28%] vs 2.74% [2.11%–6.40%], respectively; *P* < .001) and *Streptococcus oralis* (10.46% [6.61%–25.89] vs 1.54% [1.49%–2.11%]; *P* = .001) (Figure 5*A*). Conversely, nasopharyngeal samples were primarily dominated by *S pneumoniae* (median [IQR], 85.43% [47.58%–90.13%] vs 6.7% [4.81%–6.7%]; *P* < .001) (Figure 5*A*).

We also observed distinct serotype distributions between aerosol and nasopharyngeal *S pneumoniae* populations within the same individual. Among paired samples, 73.7% (14 of 19) had discordant serotypes (*P* = .009) (Figure 5*B* and Supplementary Table 4). While the difference was not statistically significant, multiple pneumococcal carriage was more common in aerosol than nasopharyngeal samples (57.9% [11 of 19] vs 26.3% [5 of 19]; *P* = .10) (Supplementary Table 4). Overall, aerosol samples exhibited greater microbial diversity than nasopharyngeal samples.

## DISCUSSION

We assessed pneumococcal carriage dynamics in otherwise healthy PLHIV-ART>1y, compared to HIV− adults. We found that PLHIV-ART>1y had a high pneumococcal carriage density and often shed MDR *S pneumoniae*. High carriage densities may contribute to the increased risk of IPD in PLHIV on ART and may also contribute to pneumococcal shedding of MDR pneumococci in the community.

Consistent with our previous findings, PLHIV-ART>1y exhibited significantly higher pneumococcal densities than HIV− adults [33]. Pneumococcal density has been associated with the onset of respiratory illnesses and severe pneumococcal pneumonia [4, 34, 35] Furthermore, in an experimental human pneumococcal carriage model, high nasal pneumococcal carriage density was linked to bacterial microaspiration into the lower respiratory tract and the activation of alveolar macrophages [36]. Moreover, PLHIV have a higher propensity than HIV− adults for harboring pneumococci intracellularly within human alveolar macrophages [37], suggesting impaired bacterial clearance associated with HIV infection. In addition, PLHIV-ART > 1 year exhibit a heightened propensity for pneumococcal carriage, associated with delayed bacterial clearance [33]. Our findings are consistent with prior studies conducted in Malawi [8, 9], South Africa [38] and Kenya [39], demonstrating increased pneumococcal carriage prevalence among PLHIV, even while on ART. While most previous work has focused on prevalence and serotype distribution, our study adds to this by characterizing phenotypic density and shedding among PLHIV on long-term ART. Taken together, these findings underscore the heightened susceptibility to pneumococcal disease among PLHIV on stable ART.

Pneumococcal transmission is thought to occur primarily through respiratory droplets [40], but direct contact through hands has also been implicated as a potential vehicle for transmission [2, 16]. We observed pneumococcal shedding (a surrogate for transmission) through nose poking and coughing during natural carriage, which was associated with non-PCV13 vaccine serotypes and vaccine escape pneumococcal serotypes 3 and 19F. Increased acquisition rates among family members have been previously associated with high pneumococcal carriage density [15]. Consistent with these findings, we demonstrate that high-density carriage in PLHIV-ART>1y is associated with an increased likelihood of pneumococcal shedding. Furthermore, a study in South Africa demonstrated that mothers with HIV transmit pneumococci more frequently to their children than HIV− mothers [41]. Moreover, insight into adult nasopharyngeal and oral pneumococcal carriage has shown that adults could be important reservoirs of pneumococcal persistent carriage in the post-PCV era [42, 43]. Collectively, by assessing pneumococcal shedding in our current study, we support the narrative that PLHIV-ART, even those on long-term ART, could serve as a source of vaccine escape serotypes, as well as serotype replacement in the community.

Furthermore, aerosol-shed pneumococci from PLHIV-ART>1y were more frequently MDR than nasopharyngeal carriage isolates. Previous studies have demonstrated that closely related streptococci species, such as *Streptococcus mitis*, a commensal of the human oropharynx, are a source of antibiotic resistance genes for *S pneumoniae* [44, 45] through horizontal gene transfer (HGT). We observed a higher prevalence of *S oralis* (a member of the *S mitis* group) in the aerosol-shed samples than in the nasopharyngeal samples. In addition, aerosol-shed samples exhibited a pneumococcal serotype population distinct from that of nasopharyngeal samples and showed trends of a higher prevalence of multiple pneumococcal carriage.

Multiple carriage has been suggested to facilitate HGT among pneumococcal isolates, potentially promoting antibiotic resistance development [46–48] Furthermore, consistent with purifying selection from daily cotrimoxazole prophylaxis in PLHIV, there was a higher prevalence of cotrimoxazole-resistant carriage pneumococci in PLHIV-ART>1y than in HIV− adults. Cotrimoxazole is a broad-spectrum antimicrobial, and resistance to it frequently occurs together with penicillin resistance among pneumococcal isolates [49, 50], which could alter the microbiota in the oral and nasopharyngeal compartments, leading to selection for MDR pneumococci. These findings suggest that the acquisition of MDR in aerosol-shed isolates among PLHIV-ART>1y could be attributed to HGT of antibiotic-resistance determinants from related streptococci species and antimicrobial-driven purifying selection.

Despite the considerable strengths of this study, including a comprehensive assessment of pneumococcal carriage dynamics in a relevant pneumococcal disease–susceptible population and high-transmission setting, it does have limitations. Although pneumococcal shedding is considered a surrogate for transmission potential [2, 16], it is not clear how often shed pneumococci result in successful transmission. Consequently, future studies should quantify the number of pneumococcal shedding events that lead to the establishment of successful transmission. Furthermore, another potential limitation of this study is that we did not assess respiratory viral coinfections, which are known to increase pneumococcal density [14, 15] and may contribute to the differences observed between PLHIV and HIV− adults.

In conclusion, our study demonstrates that PLHIV-ART>1y remain at high risk of IPD and could also be an important reservoir for shedding of MDR *S pneumoniae*. This has the potential to derail the success of the infant PCV program and our fight against AMR in settings with high HIV prevalence and a high pneumococcal disease burden, such as Malawi.

## Supplementary Material

ofaf422_Supplementary_Data

## Supplementary Data

Supplementary materials are available at *Open Forum Infectious Diseases* online. Consisting of data provided by the authors to benefit the reader, the posted materials are not copyedited and are the sole responsibility of the authors, so questions or comments should be addressed to the corresponding author.
