# Supplementary material for: High Propensity for Multidrug-Resistant Pneumococcal Shedding Among Adults Living With HIV on Stable Antiretroviral Therapy in Malawi
Source: Open Forum Infect Dis. 2025 Jul 16;12(8):ofaf422. doi: 10.1093/ofid/ofaf422 (PMC12343114; doi:10.1093/ofid/ofaf422)
Supplement: ofaf422_Supplementary_Data [file ofaf422_supplementary_data.docx]

**Supplementary materials**

**Methods**

*Definition of variables for analysis*

Pneumococcal carriage density of less than 2010 CFU/ml was considered low-density carriage, which represents values below the 25^th^ percentile (Fig. S3). Seasons are defined as Hot wet (November and April) and Cool dry (May and October) (according to Malawi Meteorological Services).

*Covariates for logistic regression analysis*

Covariates included in the models were age, sex, season, and socioeconomic status, which were selected a priori based on biological plausibility and previous literature. To identify factors influencing pneumococcal carriage prevalence and pneumococcal carriage density, the following covariates were included: HIV status, sex, age, season and socioeconomic status. To identify factors influencing pneumococcal shedding, the following covariates were included: HIV status, sex, age, season, socioeconomic status and pneumococcal carriage density. All covariates were chosen a priori based on their potential to influence pneumococcal carriage dynamics.

**Supplementary Tables**

**Table S1:** Overall baseline characteristics for all individuals enrolled

|  | **HIV-uninfected, n=54** | **PLHIV>1yr, n=90** | **p-value** |
| --- | --- | --- | --- |
| **Sex**  Female  Male | 34 (63%)  20 (37%) | 62 (69%)  28 (31%) | 0.5 **^a^** |
| **Age (Years)** | 27 (24 – 35) | 34 (26 – 38) | **0.023 ^b^** |
| **Number of ≤5 children per household**  1-year-olds  2-year-olds  3-year-olds | 38 (70%)  13 (24%)  3 (5.6%) | 77 (86%)  11 (12%)  2 (2.2%) | 0.078 **^a^** |
| **Socioeconomic status ^c^**  Median (IQR) | 7 (3 – 8) | 5 (4 - 6) | 0.11 **^b^** |
| **CD4 counts (Cells/μl)**  Median (IQR) | 786 (673 - 945) | 560 (349 - 773) | **<0.001 ^b^** |
| **ART duration (Years)**  Median (IQR) | NA | 5.5 (2.8 - 10.1) |  |
| **HIV viral load (Copies/ml) ^d^**  Median (IQR) | NA | 39 (39 - 25,150) |  |

**^a^** Pearson’s – Chi-squared test; Fisher’s exact test

**^b^** Wilcoxon rank sum test

**^c^** Socioeconomic status score based on a possession index, which is calculated as a sum of positive responses for household ownership of each of the fifteen different functioning items such as a watch, radio, bank account, iron (charcoal), sewing machine (electric), mobile phone, CD player, fan (electric), bed net, mattress, bed, bicycle, motorcycle, car, and television.

**^d^** i.e. Only 13 PLHIV>1yr had a detectable Viral load

**Table S2a:** Factors associated with non-PCV13 vaccine serotypes (NVT) carriage**.**

|  | **Univariate** | | | **Multivariable** | | |
| --- | --- | --- | --- | --- | --- | --- |
|  | **OR** | **95% CI** | **p-value** | **OR** | **95% CI** | **p-value** |
| **HIV status**  HIV-uninfected  PLHIV>1yr | -  1.53 | -  1.17 – 2.00 | **0.002** | -  1.45 | -  1.10 – 1.93 | **0.009** |
| **Sex**  Female  Male | -  0.93 | -  0.69 – 1.25 | 0.6 | -  1.00 | -  0.73 – 1.35 | >0.9 |
| **Age (Years)** | 1.02 | 1.00 – 1.03 | 0.082 | 1.01 | 0.99 – 1.03 | 0.4 |
| **Season**  Cold dry  Hot wet | -  0.93 | -  0.71 – 1.22 | -  0.6 | -  0.94 | -  0.72 – 1.24 | 0.7 |
| **Socioeconomic status ^a^**  Medium/high ses (> 3)  Low ses (≤ 3) | -  1.23 | -  0.93 – 1.61 | -  0.14 | -  1.14 | -  0.86 – 1.50 | 0.4 |

OR = Odds Ratio, CI = Confidence Interval

**^a^** Socioeconomic status score based on a possession index, which is calculated as a sum of positive responses for household ownership of each of the fifteen different functioning items such as a watch, radio, bank account, iron (charcoal), sewing machine (electric), mobile phone, CD player, fan (electric), bed net, mattress, bed, bicycle, motorcycle, car, and television.

**Table S2b:** Factors associated with PCV13 vaccine serotypes (VT) carriage

|  | **Univariate** | | | **Multivariable** | | |
| --- | --- | --- | --- | --- | --- | --- |
|  | **OR** | **95% CI** | **p-value** | **OR** | **95% CI** | **p-value** |
| **HIV status**  HIV-uninfected  PLHIV>1yr | -  1.08 | -  0.19 – 6.03 | >0.9 | -  17.0 | -  0.07 – 4095 | 0.3 |
| **Sex**  Female  Male | -  0.92 | -  0.14 – 6.23 | >0.9 | -  1.93 | -  0.21 – 18.1 | 0.6 |
| **Age (Years)** | 1.02 | 0.91 – 1.14 | 0.7 | 1.27 | 0.99 – 1.62 | 0.059 |
| **Season**  Cold dry  Hot wet | -  0.74 | -  0.53 – 1.04 | -  0.084 | -  0.67 | -  0.12 – 3.68 | 0.6 |
| **Socioeconomic status ^a^**  Medium/high ses (> 3)  Low ses (≤ 3) | -  1.05 | -  0.19 – 5.90 | -  >0.9 | -  0.55 | -  0.09 – 3.52 | 0.5 |

OR = Odds Ratio, CI = Confidence Interval

**^a^** Socioeconomic status score based on a possession index, which is calculated as a sum of positive responses for household ownership of each of the fifteen different functioning items such as a watch, radio, bank account, iron (charcoal), sewing machine (electric), mobile phone, CD player, fan (electric), bed net, mattress, bed, bicycle, motorcycle, car, and television.

**Table S3:** Pneumococcal antibiogram comparing nasopharyngeal carriage and shed pneumococci stratified by sample type among HIV-uninfected adults.

|  | **Aerosol shedding vs Nasopharyngeal carriage** | | | **Mechanical shedding vs Nasopharyngeal carriage** | | |
| --- | --- | --- | --- | --- | --- | --- |
|  | **Aerosol shedding, n=7** (%) | **Nasopharyngeal carriage, n=7** (%) | **p – value ^a^** | **Mechanical shedding, n=8** (%) | **Nasopharyngeal carriage, n=8** (%) | **p – value ^a^** |
| **Cotrimoxazole** | 6 (86) | 5 (71) | >0.9 | 6 (75) | 6 (75) | >0.9 |
| **Benzylpenicillin^b^** | 6 (86) | 4 (57) | 0.6 | 6 (75) | 6 (75) | >0.9 |
| **Tetracycline** | 6 (86) | 5 (71) | >0.9 | 8 (100) | 7 (88) | >0.9 |
| **Erythromycin** | 4 (57) | 5 (71) | >0.9 | 3 (38) | 3 (38) | >0.9 |
| **MDR** | 6 (86) | 5 (71) | >0.9 | 6 (75) | 6 (75) | >0.9 |

MDR = multi-drug resistance

**^a^** Fisher’s exact test

**^b^** i.e. Minimum inhibitory concentration using EUCAST meningitis breakpoints

**Table S4:** Sample type (aerosol shedding and nasopharyngeal carriage) stratified by participants and pneumococcal serotypes among PLHIV-ART>1yr (n=17) based on genotypic data. Highlighted in blue are similar pneumococcal serotypes in an individual from both sample types.

| **Participant** | **aerosol shedding pneumococcal serotypes** | **nasopharyngeal carriage pneumococcal serotype** |
| --- | --- | --- |
| **1** | 35A | 23A |
| **2** | 11B/11C, 20, 45 | 38 |
| **3** | 19B, 19C, 20 | 23A |
| **4** | 48 | 48 |
| **5** | 19C | 16F, 21, 29, 3, 34 |
| **6** | 21 | 21 |
| **7** | 16A, 19C, 35A | 23B1 |
| **8** | 15B/15C, 19B, 19C | 3, 34 |
| **9** | 16A, 19B, 19C, 3 | 15B/15C |
| **10 ^a^** | 19B **^a^** | 11A **^a^** |
| **10 ^a^** | 19B, 19C **^a^** | 11A **^a^** |
| **11 ^b^** | 15A, 19F **^b^** | 3 **^b^** |
| **11 ^b^** | 3 **^b^** | 3 **^b^**, 29 |
| **12** | 42, 45 | 17F, 20 |
| **13** | 45 | 34 |
| **14** | 28F | 28F |
| **15** | 07B, 07C | 07B, 07C |
| **16** | 15A, 19B, 19C, 19F | 06A |
| **17** | 16A, 19B, 19C | 4 |

**^a^** Same individual on day 3 and 21

**^b^** Same individual on day 3 and 21

**Supplementary Figures**

**Figure S1**


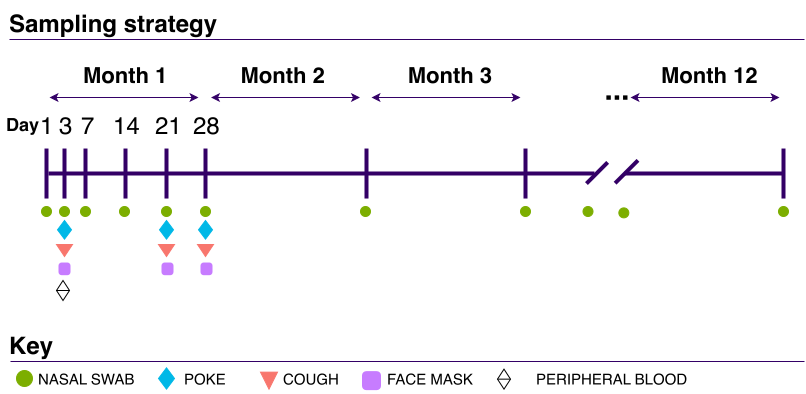


**Figure S1: Study design showing longitudinal follow-up of study participants and sample collection points for 12 months**.

**Figure S2**

**
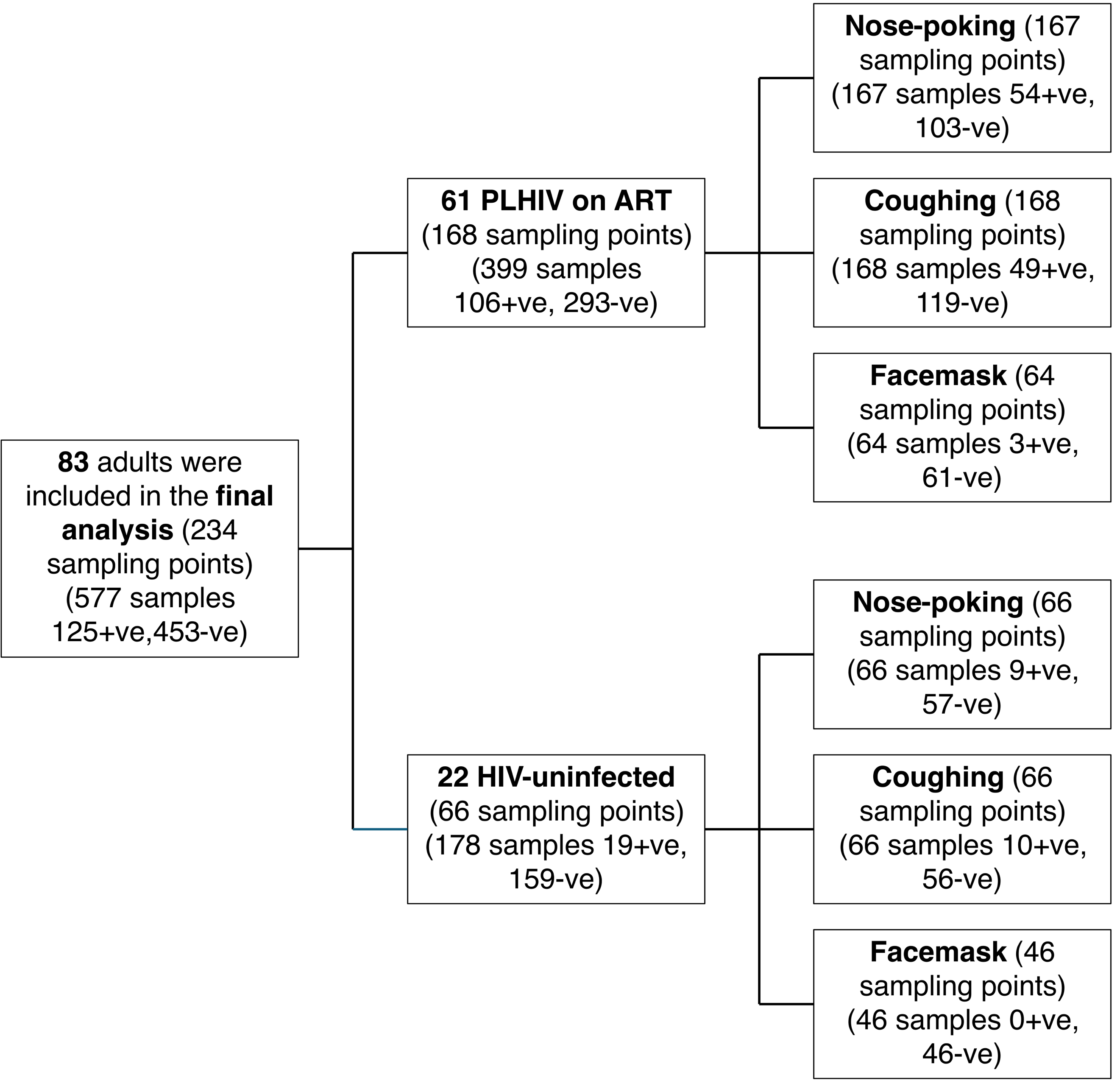
**

**Figure S2: Recruitment flow diagram for the shedding arm.** A flow diagram shows the number of adults and pneumococcal shedding samples included in the analysis among PLHIV-ART>1yr and HIV-uninfected participants**Figure S3**


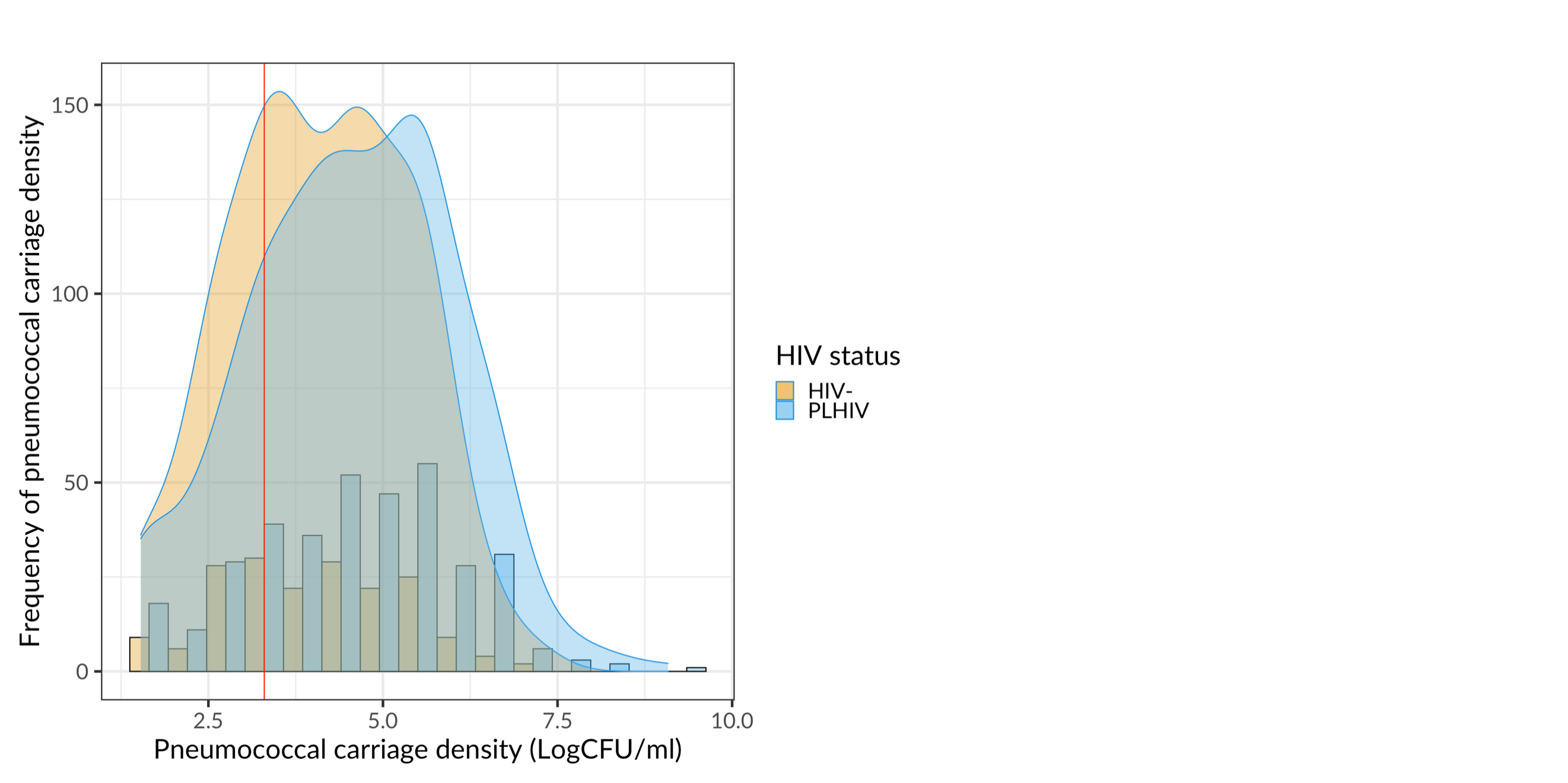


**Figure S3: Pneumococcal carriage density among PLHIV and HIV-uninfected participants.** Histogram showing the distribution of pneumococcal carriage density stratified by HIV status. The red line indicates the 25th quantile used in the multivariate analysis.
